# New Series of Hydrogen-Bonded Liquid Crystal with High Birefringence and Conductivity

**DOI:** 10.3390/molecules29143422

**Published:** 2024-07-21

**Authors:** Manel Ben Salah, Lotfi Saadaoui, Taoufik Soltani, Naoufel Ben Hamadi, Ahlem Guesmi, Ulrich Maschke

**Affiliations:** 1 Laboratoire de Physique de la Matière Molle et de la Modélisation Electromagnétique, Faculté des Sciences de Tunis, Université de Tunis El Manar, Tunis 2092, Tunisia; b.salahmanel@gmail.com (M.B.S.); taoufik.soltani@fst.utm.tn (T.S.); 2 The MOE Key Laboratory of Weak-Light Nonlinear Photonics and International Sino-Slovenian Joint Research Center on Liquid Crystal Photonics, TEDA Institute of Applied Physics and School of Physics, Nankai University, Tianjin 300457, China; 3 Chemistry Department, College of Science, Imam Mohammad Ibn Saud Islamic University (IMSIU), P.O. Box 5701, Riyadh 11432, Saudi Arabia; nabenhamadi@imamu.edu.sa (N.B.H.); amalkasme@imamu.edu.sa (A.G.); 4 Unité Matériaux et Transformations (UMET), UMR 8207–CNRS, University Lille, CNRS, INRAE, Centrale Lille, F-59000 Lille, France

**Keywords:** liquid crystal, nematic phase, conductivity, birefringence, hydrogen-bonding

## Abstract

Liquid crystals with high dielectric anisotropy, low operational thresholds, and significant birefringence (Δ*n*) represent a key focus in soft matter research. This work introduces a novel series of hydrogen-bonded liquid crystals (HBLCs) derived from 4-n-alkoxybenzoic, 4-alkoxy-3-fluorobenzoic derivatives (nOBAF), 4-alkoxy-2,3-fluorobenzoic derivatives (nOBAFF), and 2-fluoro-4-nitrobenzoic acid. The HBLCs were characterized using Fourier transform infrared spectroscopy, and their thermal behavior was evaluated via differential scanning calorimetry. Optical observations were conducted using polarized optical microscopy. The results indicate that mixtures containing benzoic acid with a bilateral fluorine substituent exhibit both SmA and SmC phases, while those with a unilateral fluorine substituent exhibit nematic and SmA phases. Moreover, an increase in the length of the alkoxy chain results in an expanded mesophase temperature range. This study demonstrates that the presence of a fluorine substituent and the incorporation of an NO_2_ group in the molecular structure result in an increase in dielectric permittivity, DC conductivity, dielectric anisotropy, and birefringence.

## 1. Introduction

Liquid crystals (LCs) represent a fascinating branch of self-assembled soft condensed matter that has attracted considerable attention in fundamental sciences and display applications. A variety of LC compounds with distinct structural characteristics have been synthesized using diverse substrates, which have been extensively examined for their potential applications [1,2,3,4,5,6,7,8,9,10,11]. Research has demonstrated that the shape of the molecule plays a critical role in determining the formation of the mesophase [1,4,8]. A number of distinct phases have been identified, including nematic [2,3,6,12], smectic [1,2,11,12,13,14], twisted nematic [4,8,9], ferroelectric [14], and antiferroelectric [7] phases. For industrial applications, it is desirable to have LCs with excellent electro-optic properties. However, the electro-optic performance is significantly influenced by birefringence, dielectric anisotropy, or rotational viscosity. For instance, high dielectric anisotropy allows for the fabrication of LC displays with reduced threshold voltages. Although numerous mesogens with large dipole moments have been synthesized, they generally contain cyano (CN) and nitro (NO_2_) groups [15,16,17,18,19]. For example, n-CyanoBiphenyl compounds exhibit positive dielectric anisotropy within the range of (8–12) [15,16]. Recent studies have demonstrated that a significant number of elongated molecules containing NO_2_ or CN, with dipole moments of approximately 12 D, exhibit stable ferroelectric nematic phases. This phase typically exhibits low threshold voltages (approximately 0.4V) [18] and high dielectric constants (10^4^–10^5^) [20,21,22].

Furthermore, display applications necessitate nematic LCs with a high birefringence [23,24,25,26,27] to ensure optimal performance across a wide range of spectral regions, including visible light [23], near infrared (NIR) [24], mid infrared (MIR) [25], millimeter wave [26], and terahertz [27]. LC mesogens have been developed by incorporating the acetylene group (C≡C) into the mesogenic segment, extending the π-electron conjugation of the molecule structure and allowing for high birefringence. However, these materials typically exhibit relatively high rotational viscosity and melting points. Various attempts have been made to reduce these parameters, including the introduction of unsaturated alkene groups, such as alkyloxy or but-3-enyl, into the LC molecules. This has been performed in order to facilitate the formation of nematic phases with lower rotational viscosity [3,28].

It is well-established that the inclusion of a fluorine atom into a molecule can significantly alter its physical and chemical properties due to its high electronegativity, low polarizability, and strong bond strength. Consequently, the lateral fluorine substituent has been employed to reduce the rotational viscosity and the melting point of LCs [28,29,30,31]. In addition, this substituent also expands the temperature range of the nematic phase [8]. Moreover, the impact of lateral substituents is contingent upon the number and position of the fluorine atom. Researchers have developed fluorinated calamitic mesogens that exhibit broad nematic temperature ranges and low melting points. Dabrowski et al. demonstrated that replacing hydrogen atoms with fluorine destabilizes the SmE phase and leads to a highly birefringent nematic phase [29]. The incorporation of a lateral fluorine substituent in LCs enhances the optical, electrical, and the temperature range of the nematic phase, rendering it an attractive candidate for photovoltaic applications.

Significant research has been conducted with the objective of designing and synthesizing hydrogen-banded LCs (HBLCs) and helical ferroelectric liquid crystal for applications in GHz [32] and THz [33,34] frequency domains. In particular, the potential of dichroism-free HBLC as an optical material for THz devices was demonstrated [33] in comparison to those using dichroic LC materials. The relationship between the molecular structures of LCs and their properties was elucidated, as demonstrated by the references [12,13,26,32,33]. It is of particular importance to note that such HBLCs possess a high degree of polymorphism, displaying both smectic and nematic phases. It has been reported that HBLCs exhibit low anisotropy and high driven voltage [31,33,35], which highlights the need to improve these parameters. Consequently, the magnitude of the birefringence (Δ*n*) and the dielectric anisotropy (Δ*ε*) can be chemically controlled by the introduction of polar groups, such as NO_2_ and CN groups, into the mesogen core.

In this report, HBLCs mixtures with varying molecular shape anisotropy will be examined. The bi-component mixtures contain one compound with a fluorine and a NO_2_ group, while the second one possesses either zero, one, or two fluorine atoms. This study specifically examines the effects of position and number of fluorine atoms of the second compound on the thermal, dielectric, and electro-optic properties of the HBLC mixtures.

## 2. Results 

### 2.1. FTIR Analysis

**Figure 1:**
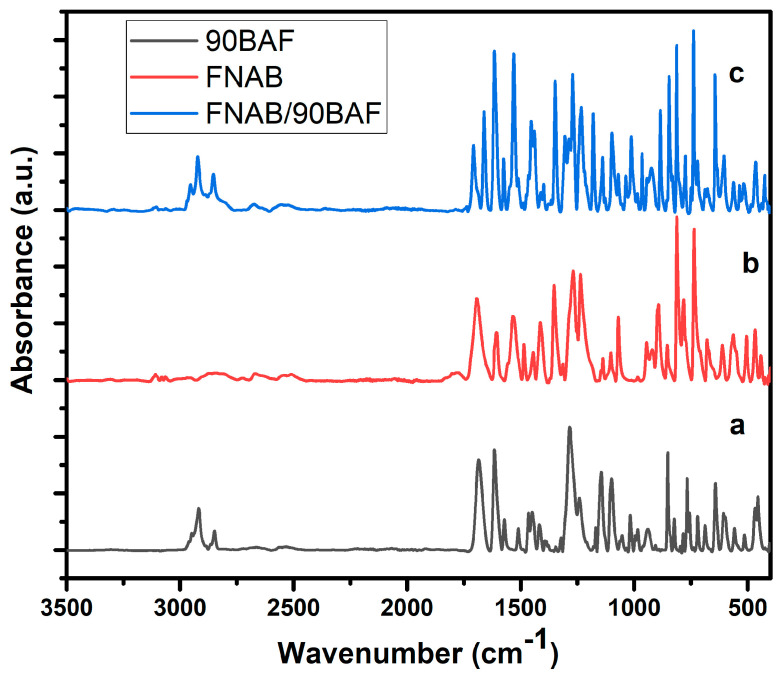
FTIR of supramolecular H–bonded compounds: (**a**) FNBA, (**b**) 9OBAF, and (**c**) the FNBA/9OBAF mixture.

In order to gain further insight at the molecular level, FTIR spectroscopy was employed. The FTIR spectra of 9OBAF, FNBA, and FNBA/9OBAF are presented in Figure 1a–c, respectively.

The spectra of 9OBAF and FNBA/9OBAF exhibit a broad band at 2700–3300 cm^−1^, which is assigned to the ν(O–H) mode of carboxylic acid groups. In addition, the ν(C=O) mode was observed in the form of sharp bands at 1683 cm^−1^, 1694 cm^−1^, and 1703 cm^−1^ for 9OBAF, FNBA, and FNBA/9OBAF, respectively. The observed differences in the wavenumbers of the three compounds in conjunction with the absence of absorbance at 3500 cm^−1^, which is characteristic of the free O–H group, provide compelling evidence that the FNBA and 9OBA molecules are hetero-associated through hydrogen bonding between the carboxylic acid groups. It is also noteworthy that a comparable outcome was observed for the other synthesized compounds.

### 2.2. Phase Behavior

**Figure 2:**
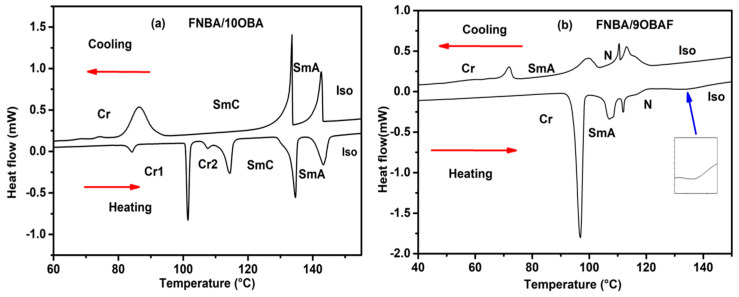
DSC thermograms of (**a**) the FNBA/10OBA mixture and (**b**) the FNBA/9OBAF blend.

The phase transition temperatures and the temperature range of the LC phase of the compounds under study were investigated using DSC and POM techniques. As an illustration, DSC analysis and POM texture investigations were presented for FNBA/10OBA and FNBA/9OBAF mixtures. The DSC thermogram of FNBA/10OBA displays four endothermic peaks upon heating and three exothermic peaks upon cooling, which correspond to the presence of two mesophases (Figure 2a).

**Figure 3:**
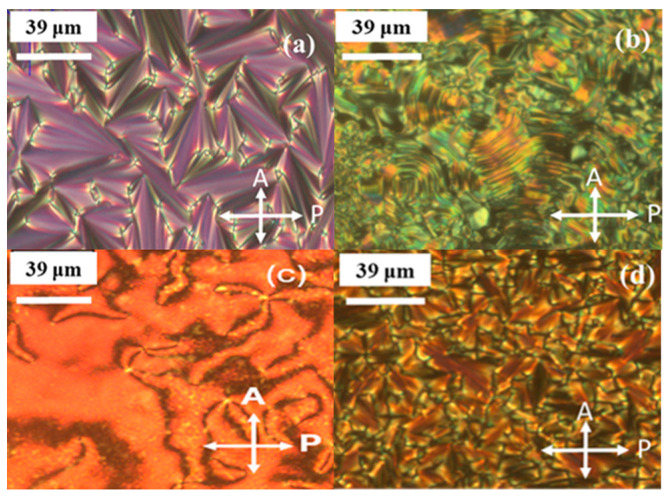
Textures observed by POM upon cooling: (**a**) SmA and (**b**) SmC phases for the FNBA/10OBA mixture and (**c**) nematic and (**d**) SmA phases for the FNBA/9OBAF blend.

Upon cooling from the isotropic phase, this compound exhibits the conical focal texture of SmA and the fan-shaped textures of SmC, as illustrated in Figure 3a,b. Furthermore, the heating scan indicates that the mixture FNBA/9OBAF melts at 96 °C and transitions to an isotropic phase at 132 °C (see Figure 2b). The mixture exhibits two liquid crystal (LC) mesophases, as identified by polarizing optical microscopy (POM) in Figure 3c,d. Upon cooling, the temperature range of [T_Iso-N_ − T_N-SmA_ = 115.4 − 101 °C] displays the nematic phase with a Schlieren texture, whereas the temperature range of [T_N-SmA_ − T_SmA-Cr_ = 101 − 71.6 °C] exhibits the SmA phase characterized by a conic focal texture. Table 1 presents the mesomorphic transition temperatures (in degrees Celsius) and enthalpy (in Joules per gram) upon cooling. The phase transitions of the FNBA/14OBAF mixture are analogous to those of the FNBA/9OBAF mixture. In comparison to FNBA/9OBAF, the temperature of crystallization is observed to decrease, while the Iso-N phase transition temperature increases by 6 °C. This results in an expanded temperature range of the LC phase. It should be noted that the occurrence of superimposed thermal peaks at the SmA-N phase transition is a consequence of the coexistence of nematic and SmA phases, as evidenced by POM observations.

### 2.3. Dielectric Properties

**Figure 4:**
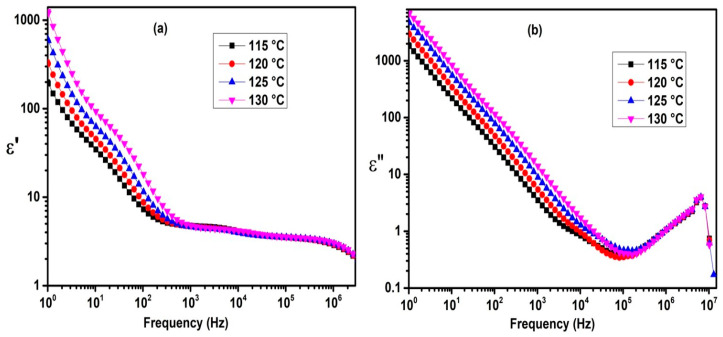
Frequency dependence of *ε*′ (**a**) and *ε*″ (**b**) for the FNBA/10OBA blend at several temperatures.

Figure 4 illustrates the frequency dependence of the real (*ε*′) and imaginary (*ε*″) parts of the complex permittivity at different temperatures for the FNBA/10OBA system, as an example.

**Figure 5:**
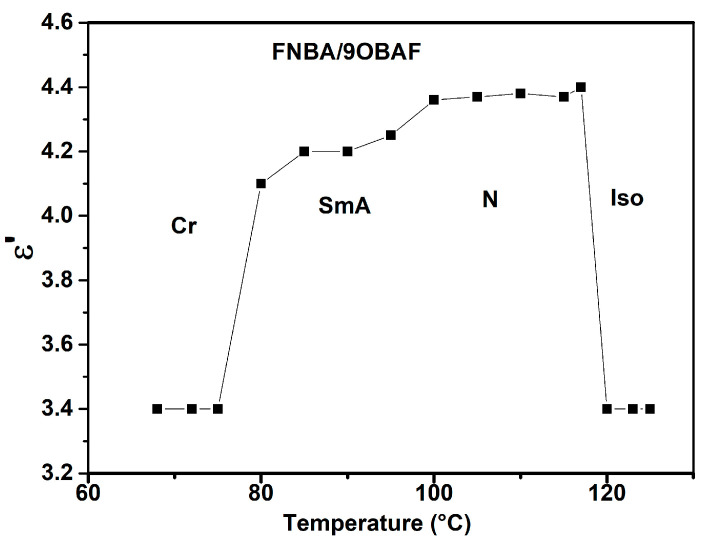
Temperature dependence of *ε*′ for the FNBA/9OBA mixture at 10 kHz.

At frequencies below 1000 Hz, the real (*ε*′) and imaginary (*ε*″) parts of the permittivity decrease as the frequency increases, due to the ionic contribution. In the higher frequency regime, *ε*′ becomes nearly constant, corresponding to the static dielectric constant of the LC. However, the imaginary part of the complex permittivity (*ε*″) exhibits the soft mode (fluctuations of the short molecular axis), which is characteristic of the smectic and nematic phases [36,37]. In addition, the present data show relatively high *ε*″ values as compared to the dielectric results obtained for 10OBAFF/8OBA [31], which are due to the presence of the high polar NO_2_ group. Dielectric spectroscopy is a valuable tool for studying the phase transitions of the LCs [38,39]. Figure 5 illustrates the effect of temperature on the dielectric spectra by plotting *ε*′(T) for FNBA/90BAF. In the isotropic phase, *ε*′ remains constant during the cooling process and then increases significantly to reach its maximum value at the Iso-N phase transition. In the nematic phase, the dielectric constant (*ε*′) remains relatively constant and does not vary significantly with the temperature. Discontinuities were observed at the N-SmA and SmA-Cr phase transitions, which are consistent with the findings in [37].

Conversely, the substantial enhancement in *ε*′ and *ε*″ at low frequencies due to the ionic contribution indicates the potential occurrence of ionic diffusion phenomena. This can be described by the following equation [16]:

(1) $$\varepsilon^{\prime }=\frac{nq^{2}D^{\frac{3}{2}}}{\pi^{\frac{3}{2}}\;\varepsilon_{0}dk_{B}T}f^{\frac{-3}{2}}+\varepsilon_{b}$$

(2) $$\varepsilon^{\prime \prime }=\frac{nq^{2}D}{\pi \varepsilon_{0}k_{B}T}f^{-1}$$

In these equations, *q* represents the electric charge, *d* stands for the cell gap, *ε*_0_ is the permittivity in free space, *k_B_* represents the Boltzmann constant, *T* stands for the absolute temperature, *n* is the bulk ionic concentration, and *ε_b_*′ represents the intrinsic dielectric constant of the LC bulk.

To extract the ionic concentration (*n*_*ion*_) and the diffusion coefficient *D*, the spectra of *ε*′ and *ε*″ were fitted together in the range of 1–5 kHz using Equations (1) and (2). Table 2 presents the values for the diffusion constant *D*, the ionic concentration *n*_*ion*_, and the mobility of free ions *µ*. The latter quantity was calculated using the diffusion coefficient *D* [38].

(3) $$\mu =\frac{q}{k_{B}T}D$$

As anticipated, an elevation in temperature is accompanied by an enhancement in all parameters. The rise in the ionic concentration at elevated temperatures can be attributed to the thermal energy acquired by the ions, which enables them to detach from the LC molecules and become mobile. The rise in mobility and *D* is attributable to the reduction in viscosity at higher temperatures.

The measurement of conductivity represents a reliable method for describing the ionic behavior in a sample, as it is proportional to the concentration of space charge [40]:

(4) $$\sigma =\varepsilon_{0}\omega \varepsilon^{\prime \prime }$$

The horizontal part of the curve for the SmA and nematic phases represents DC electric conductivity. This value was derived through a nonlinear fitting of the *σ*_AC_ plot using frequency to the universal power law [40]:

(5) $$\sigma =\sigma_{DC}(1+{\left(\frac{f}{f_{c}}\right)}^{m})$$

where *σ_DC_* is the *DC* conductivity, *f*_*c*_ stands for the characteristic frequency, and *m* represents the degree of interaction between the mobile ions and their surroundings.

**Figure 6:**
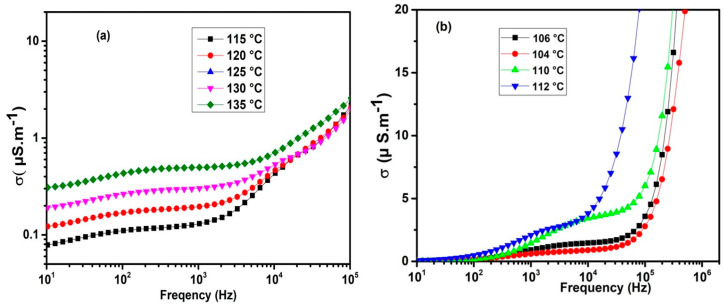
Plot of the frequency dependence of the conductivity for the (**a**) FNBA/10OBA and (**b**) FNBA/9OBAF mixtures.

It is anticipated that an increase in *σ_DC_* will be observed as a consequence of the temperature-dependent nature of both the ionic concentration and mobility. This is because *σ_DC_* is directly proportional to these parameters. It was observed that the values of *σ_DC_* in FNBA/9OBAF are higher than those in FNBA/10OBA (see Figure 6). This can be attributed to the higher mobility in the former mixture. Furthermore, these mixtures demonstrate a high level of DC conductivity in comparison to other calamitic LCs [38]. This can be explained by the presence of molecules with high dipole moments along their molecular long axes, influenced by polar terminal groups (NO_2_), fluoro substituents, and ester groups. Furthermore, a comparison of the *σ_DC_* values of FNBA/9OBAF and FNBA/10OBA reveals that the lateral fluoro atom, which facilitates the delocalization of π-electrons in the aromatic group, plays a pivotal role in the enhancement of this parameter. As reported by Manabe et al. [22], a high longitudinal dipole moment and a lateral fluoro substituent lead to the appearance of high dielectric permittivity. Based on these considerations, it appears that a large dipole moment in the long axis of a rod-like molecule and the lateral fluoro substituent are of primary importance for the appearance of high conductivity. Another contributing factor is the degree of molecular order, which improves ionic mobility and, therefore, the conductivity.

### 2.4. Properties of the Nematic Phase

**Figure 7:**
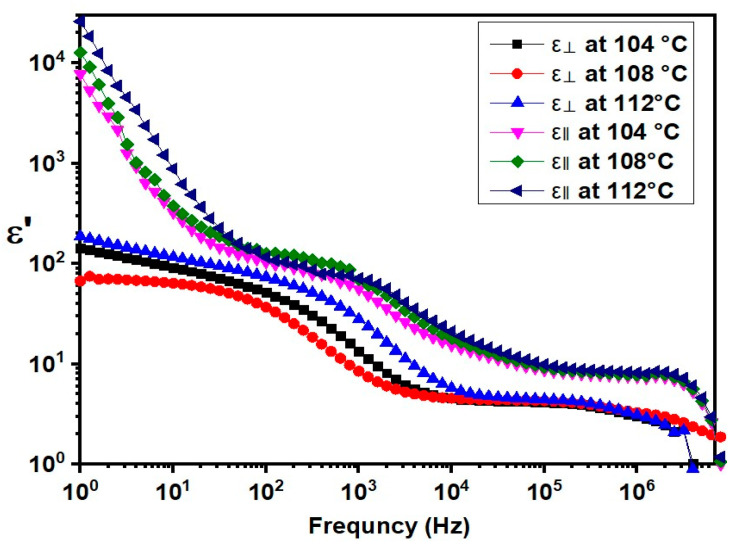
Frequency dependence of (*ε*’_⏊_) and (*ε*’_‖_) for the FNBA/9OBAF blend at several temperatures.

Figure 7 shows the real component (*ε*′) of the FNBA/9OBAF in planar (*ε*_⏊_) and homeotropic (*ε*_‖_) alignments over a frequency range of [1 Hz–10 MHz]. At lower frequencies, there is a significant decrease in *ε*′ as the frequency increases due to the ionic contribution, which is analogous to that observed in FNBA/10OBA. At frequencies ranging from 10^3^ to 10^5^ Hz, *ε*′ remains nearly constant, representing the static permittivity. At this range, ionic impurities are no longer able to follow the periodic inversion of the electric field. The values of *ε*_‖_ are higher than those of *ε*_⏊_, indicating a positive dielectric anisotropy.

**Figure 8:**
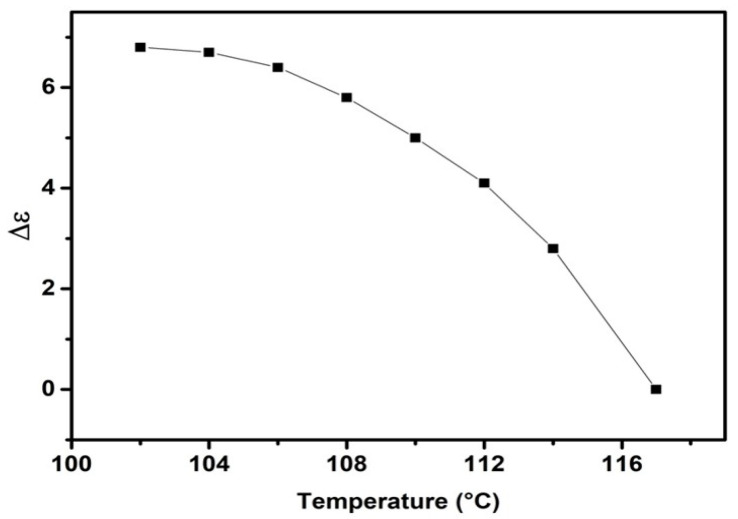
The variation in the dielectric anisotropy for the FNBA/9OBAF mixture as a function of the temperature.

Figure 8 illustrates the variation in dielectric anisotropy with temperature. The graph indicates a decrease in dielectric anisotropy with increasing temperature, with a significant decrease occurring at the Iso-N phase-transition temperature. The obtained Δ*ε* values are 4.6, 5.3, and 6.5 at 112 °C, 108 °C, and 104 °C, respectively, indicating that the target compounds FNBA/9OBAF possess high dielectric anisotropy. Furthermore, when compared to the dimeric 9OBAF (Δ*ε* = 0.6), the present compound exhibits a significantly larger Δ*ε* value. The presence of the NO_2_ group in these compounds is responsible for their non-symmetric configuration, increased dipole moment, and higher polarizability as compared to the dimeric compound. Consequently, a larger number of molecules align longitudinally, increasing the dielectric anisotropy.

**Figure 9:**
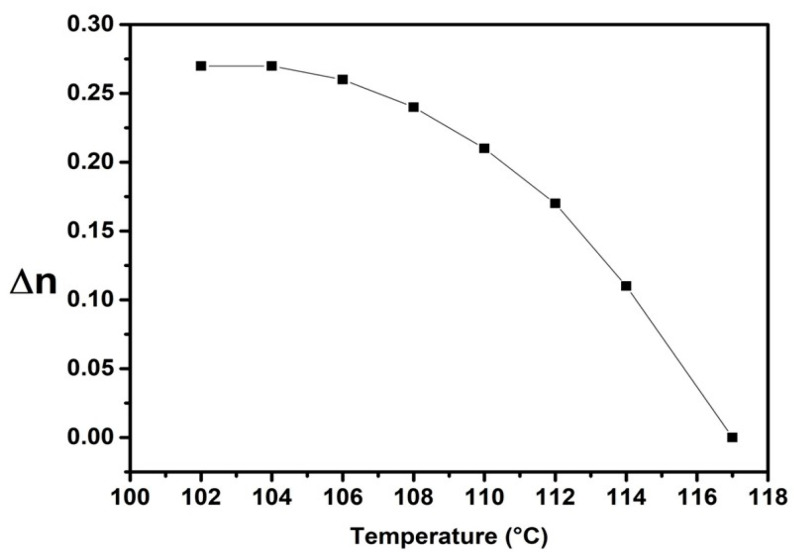
Birefringence as a function of temperature for the FNBA/9OBAF mixture.

The optical anisotropy, or birefringence (Δ*n*), is defined as the difference between the ordinary index (*n*_o_) and the extraordinary index (*n*_e_): Δ*n* = *n*_e_ − *n*_o_. This parameter is a crucial physical property of HBLCs and plays an essential role in their applications. Figure 9 illustrates the temperature dependence of Δ*n*, which exhibits a slight decrease over the nematic range with increasing temperature and a rapid decrease near the Iso-N phase transition. The curve illustrates a high Δ*n* value for FNBA/9OBAF. This compound exhibits higher Δ*n* values (0.27 at 104 °C) as compared to the well-known 5CB (Δ*n* = 0.17) and E7 (Δ*n* = 0.24) [41] and the dimeric compounds nOBAF (Δ*n* = 0.2), while it is lower than that obtained in tolane LCs (0.3–0.4) [29]. This indicates that the incorporation of the NO_2_ group and fluorine substituent in the molecular structure enhances the Δ*n* value of the LC.

**Figure 10:**
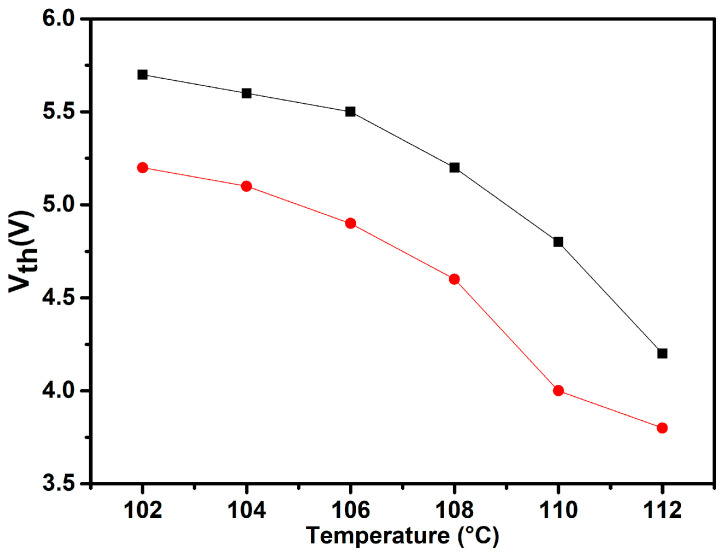
Temperature dependence of the threshold voltage for the FNBA/9OBAF blend, obtained through two distinct methods: POM observations (■) and capacitance measurements (●).

The threshold voltage (*V_th_*) is defined as the voltage required to induce the Freedericksz transition. Figure 10 depicts the variation in *V_th_* as a function of temperature. The synthesized FNBA/9OBAF blend exhibits threshold voltages between 4.2 and 5.7 V and between 3.8 and 5.2 V, respectively. The former values were achieved when the color of the texture began to change. The latter values were obtained from capacitance measurements. The latter values were found to be approximately 1 V lower than those obtained by POM observations across the entire investigated temperature range. It should be noted that the obtained threshold voltages from the FNBA/9OBAF blend were lower than those from the other HBLC material previously reported in the literature [31,33,35]. For example, the dimeric compound presents a higher threshold voltage (6–7 V) [31,35]. The enhanced dielectric anisotropy is responsible for the relatively elevated *V_th_*, as described by the following equation [15]:

(6) $$V_{th}=\pi \sqrt{K_{1}/\left(\varepsilon_{0}∆\varepsilon \right)}$$

It is crucial to acknowledge that comparable data have also been documented by Yamaguchi [33].

### 2.5. Polymer Dispersed Liquid Crystals

**Figure 11:**
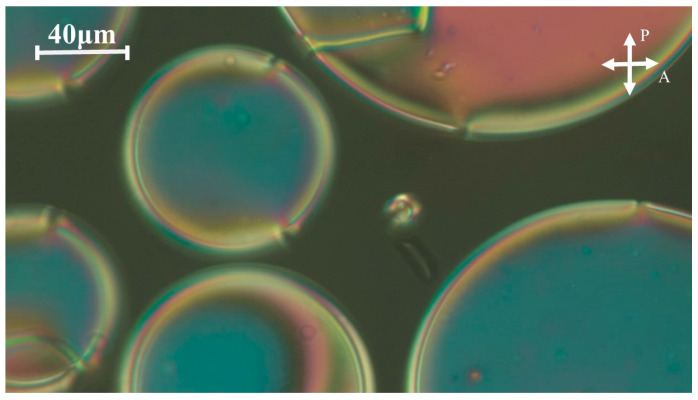
POM image of a PDLC film revealing a polystyrene matrix (40 wt-%) and a FNBA/9OBAF HBLC blend (60 wt-%), which form phase-separated liquid crystalline domains.

In order to demonstrate one of the potential applications of the synthesized HBLC mixtures, polymer-dispersed liquid crystal (PDLC) samples were prepared. PDLCs have garnered significant interest due to their versatile electro-optical properties, which render them suitable for a wide array of applications [42,43,44,45,46]. Figure 11 illustrates an image from the POM observations of a PDLC film elaborated by the thermally induced phase separation of FNBA/9OBAF as a HBLC blend with styrene as a monomer. The image demonstrates the presence of a phase-separated sample morphology, revealing a polystyrene matrix phase-separated from liquid crystalline domains consisting of a FNBA/9OBAF HBLC blend.

However, because the electric field responsible for reorienting the LC molecules is inversely proportional to the radius (*R*) of the HBLC domains, a radius of several tens of micrometers, as illustrated in Figure 11, would result in a relatively low electric field. A straightforward formula for assessing the reorienting field is provided by the following equation:

(7) $$E=\frac{1}{R}\sqrt{\frac{K}{\varepsilon_{0}∆\varepsilon }}$$

Given the high dielectric anisotropy, the estimated reorienting electric field is relatively low.

## 3. Materials and Methods

### 3.1. Preparation of Mixtures

2-fluoro-4-nitrobenzoic acid (FNBA, purity 98%) and 4-decyclobenzoic acid (10OBA, purity > 98%) were acquired from Sigma-Aldrich, Schnelldorf, Germany. HPLC-grade dimethylformamide (DMF, purity 99.5%) was purchased from Fisher Scientific, Fisher Scientific, Pittsburgh, PA, USA). The synthesis and properties of 4-alkoxy-3-fluorobenzoic (nOBAF) acid and 4-alkoxy-2,3-fluorobenzoic acid (nOBAFF) have already been reported in [31,35].

**Figure 12:**
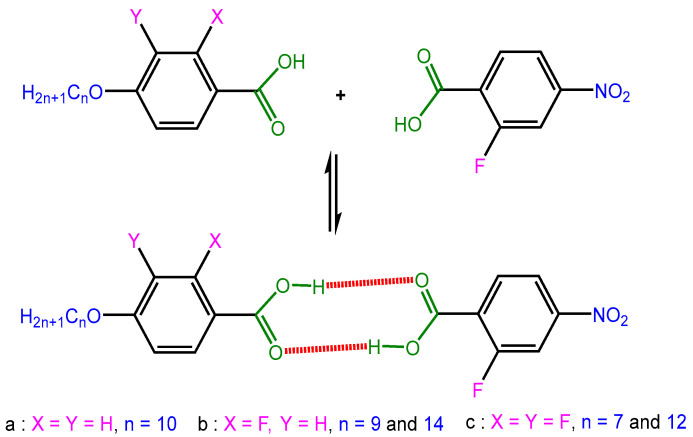
Possible interactions between FNBA and benzoic acid or fluorinated benzoic acid (10OBA, nOBAF, and nOBAFF). Red color show the H–bound.

The HBLC mixtures FNBA/10OBA, FNBA/9OBAF, FNBA/14OBAF, FNBA/7OBAFF, and FNBA/12OBAFF were prepared by dissolving a blend of FNBA and either benzoic acid or fluoro-benzoic acid in a 1:1 molar ratio in DMF. After thorough mixing, the sample was allowed to cool slowly in order to evaporate the ethanol completely until the sample mass became constant. The resulting powders were subjected to vacuum drying for a minimum of 20 h prior to use. Figure 12 depicts the structures of these compounds.

### 3.2. Measurement Set-Up and Instruments

Fourier transform infrared spectra were recorded on the dry powders in attenuated total reflectance mode (FTIR-ATR) using a Nicolet™ iS50 FT-IR spectrometer (THORLABS, Munich, Germany). The spectra were recorded in the range of 4000–400 cm^−1^ at a resolution of 4 cm^−1^ and using 32 scans for each spectrum. A differential scanning calorimeter (DSC) was utilized to perform the calorimetric measurements, with a nitrogen purge gas applied. The Perkin–Elmer DSC 7 apparatus was employed to perform the cooling and heating cycles at a rate of 3 °C per minute. The DSC measurements were performed on samples with a mass of approximately 3–4 mg. The enthalpies and transition temperatures were determined using the Perkin–Elmer Pyris software version 13.1.1. Polarized optical microscopy (POM) was conducted using an Olympus BX50 polarized optical microscope, equipped with a digital CCD camera (Sony XCD-U100CR). In order to investigate the thermal, electro-optic, and dielectric behavior, the compounds were filled by capillarity in the isotropic phase into commercially available planar alignment cells (EHC from Japan) with a thickness of 5.5 µm and an active area of 0.25 cm^2^. The dielectric permittivity (ε* = *ε*′ − *iε*″) of the HBLC mixtures was determined using an impedance/gain phase analyzer (Solartron SI1260) coupled to a 1296 dielectric interface in the frequency range from 1 Hz to 10 MHz. The parallel permittivity (*ε*_‖_) was determined using a homeotropic cell, while the perpendicular permittivity (*ε*_⏊_) was measured under an AC voltage of 0.5 V. Consequently, the dielectric anisotropy Δ*ε*, defined as the difference between the parallel and perpendicular permittivities, *ε*_‖_ and *ε*_⏊_, respectively, could be estimated.

The temperature of the sample was meticulously regulated to within a margin of ±0.1 °C through the utilization of a Linkam TMS 94 apparatus. To assess the electro-optic responses of the HBLC mixtures, an electric voltage was applied to the cell, which was connected in a series to an external electric resistance (1 kΩ). Subsequently, the sample was positioned between crossed polarizers under a polarizing microscope. An Agilent 33220A waveform function generator was employed to apply the electric voltage, which could be either a positive direct current (DC) or a sinusoidal signal with adjustable amplitude and frequency. The birefringence Δ*n* was calculated using the phase difference *δ*, as given by the following formula:

(8) δ=2πd Δn/λ

where *d* is the cell gap, and *λ* = 546 nm represents the wavelength.

The parameter *V_th_* is of particular importance in the context of LCs, as it represents the minimum voltage value required to reorient LC molecules. In the present case, the value of *V_th_* was obtained through a process of observation and measurement. The observations were made using a polarizing optical microscope (POM) [36], while the measurements were taken using an Agilent LCR meter (E4980A). The frequency of the applied voltage, which ranged from 0 to 15 V, was 1 kHz. The specifics of this procedure have been delineated in [37].

Polymer-dispersed liquid crystal (PDLC) films were prepared using the thermally induced phase separation technique, which involved a prepolymer mixture comprising a styrene monomer, FNBA/9OBAF, and azobisisobutyronitrile (AIBN) as the initiator. The purified and freshly prepared monomer (40 wt-%) was combined with the HBLC blend (60 wt-%) and a catalytic amount of AIBN, resulting in the formation of an optically homogeneous prepolymer mixture. Subsequently, the mixture was injected into 10-µm-thick ITO-coated LC cells (HG cells from AWAT, Warsaw, Poland) via capillary action and heated to 70 °C. This controlled heating process enables the precise regulation of the polymerization reaction, resulting in the formation of well-dispersed LC domains within the polystyrene matrix.

## 4. Conclusions

In conclusion, new fluorinated HBLCs derived from 4-n-alkoxybenzoic acid (10OBA), 2-fluoro-4-nitrobenzoic acid (FNBA) and lateral fluorine-substituted derivatives (nOBAF and nOBAFF) have been successfully analyzed. The investigated mixtures exhibited a diverse array of mesophases, including SmA and nematic phases, contingent on the composition of the HBLC mixture, including the length of the alkoxy chain and the number of fluorine atoms. The structural variations and composition significantly impacted the temperature range of the mesophase. It is noteworthy that the HBLCs under investigation exhibited high dielectric permittivity and DC conductivity. Moreover, the nematic phases exhibited high birefringence and dielectric anisotropy, which can be attributed to increased molecular polarizability and dipole moment. This study emphasizes the significance of molecular design and composition in tailoring the properties of HBLCs for specific applications. In particular, it highlights the potential of enhancing optical, electrical, and thermal characteristics relevant to advanced materials and device technologies through the manipulation of molecular composition.

## Figures and Tables

**Table 1:** Mesomorphic transition temperatures and enthalpies (in parenthesis) upon cooling.

Substances	Transition Temperatures (°C) and Enthalpy (J/g)
FNBA/10OBA	Cr 86 (49.2) SmC 133.4 (37.2) SmA 142 (22.7)Iso
FNBA/9OBAF	Cr 71.6 (18.1) SmA 101 (13.5) N 115.4 (17.4) Iso
FNBA/14OBAF	Cr 70.4 (61.5) SmA 109 (24.3) N 121.5 (12.6) Iso
FNBA/7OBAFF	Cr 79.3 (51.2) SmA 104 (6.4) N 120.9 (7.3) Iso
FNBA/12OBAFF	Cr 70 (78.3) SmA 99.5 (1.7) N 112 (1.3) Iso

**Table 2:** Electro–physical parameters of the studied compounds.

	*D* (m^2^s^−1^)	*n* (m^−3^)	*µ* (m^2^s^−1^V^−1^)
FNBA/10OBA (106 °C)	6.1 × 10^−^^11^	9.6 × 10^19^	1.43 × 10^−^^9^
FNBA/10OBA (112 °C)	4.3 × 10^−^^11^	2.3 × 10^20^	1.8 × 10^−^^9^
FNBA/10OBA (106 °C)	4.1 × 10^−^^10^	1.8 × 10^19^	8.1 × 10^−^^9^
FNBA/10OBA (112 °C)	7.6 × 10^−^^10^	2.5 × 10^19^	9.4 × 10^−^^9^

## Data Availability

Data are contained within the article.
